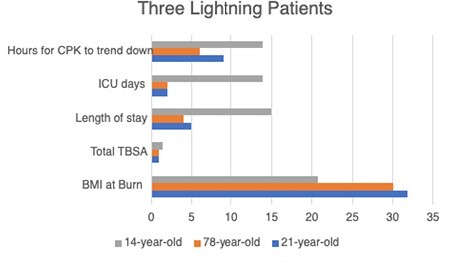# 793 Lightning Burns and General Recommendations

**DOI:** 10.1093/jbcr/irae036.334

**Published:** 2024-04-17

**Authors:** Shreya Arora, Jaynie X Criscione, Bilal Koussayer, Marian Mikhael, Ellie Randolph, Nicole K Le, Kristen Whalen, Kristina Buller, Jared Troy, Jake Laun

**Affiliations:** Morsani College of Medicine USF, Tampa, FL; University of South Florida, Tampa, FL; University of South Florida, Virginia Beach, VA; Morsani College of Medicine USF, Tampa, FL; University of South Florida, Tampa, FL; University of South Florida, Virginia Beach, VA; Morsani College of Medicine USF, Tampa, FL; University of South Florida, Tampa, FL; University of South Florida, Virginia Beach, VA; Morsani College of Medicine USF, Tampa, FL; University of South Florida, Tampa, FL; University of South Florida, Virginia Beach, VA; Morsani College of Medicine USF, Tampa, FL; University of South Florida, Tampa, FL; University of South Florida, Virginia Beach, VA; Morsani College of Medicine USF, Tampa, FL; University of South Florida, Tampa, FL; University of South Florida, Virginia Beach, VA; Morsani College of Medicine USF, Tampa, FL; University of South Florida, Tampa, FL; University of South Florida, Virginia Beach, VA; Morsani College of Medicine USF, Tampa, FL; University of South Florida, Tampa, FL; University of South Florida, Virginia Beach, VA; Morsani College of Medicine USF, Tampa, FL; University of South Florida, Tampa, FL; University of South Florida, Virginia Beach, VA; Morsani College of Medicine USF, Tampa, FL; University of South Florida, Tampa, FL; University of South Florida, Virginia Beach, VA

## Abstract

**Introduction:**

The odds of being struck by lightning are one in a million, however lightning strike accounts for 24,000 annual fatalities worldwide (Jensen et al, 2023). Due to its rarity, guidelines for the management of burns acquired by lightning strike are scarce. Current standard of treatment is to treat burns acquired as one would treat electrical burns (Stander & Wallace, 2011). We present a case series of three patients that were struck by lightning and contrast their outcomes to patients with electrical burns. We attempt to provide general recommendations for lightning burns.

**Methods:**

We conducted retrospective chart review of patients presenting between 2015- 2022 for electrical and lightning burns. SPSS V28 was used for statistical analysis. Lightning burn patients were matched to electric burn patients by age, gender, Total Burn Surface Area (TBSA), and comorbidities. Descriptive statistical analysis was conducted between the three groups due to discrepancies in sample size.

**Results:**

We report three lightning burn cases: 2 adult males with 1-1.5% TBSA second-degree burns and 1 pediatric case with 4% TBSA. The pediatric case had a more complex hospital course: longer stay (15 vs 4-5 days), ICU stay (13 vs 2 days), and required ventilation but no surgery.

In adults aged 19-29, electrical burns patients had longer hospital stays (6.38 vs 5.00 days), similar ICU stays (2.25 vs 2 days), and lower initial CPK (1624.29 vs 3506.00) than lightning burn patients. In those 50+ with TBSA < 5% and hypertension, electrical burns patients had longer stays (6.75 vs 4 days), longer ICU stays (3.25 vs 2.00 days), and lower initial CPK levels (435.5 vs 648.00). CPK normalization took longer for electric burns (29.5 vs 6 hours). In younger patients, lightning burns had lower initial myoglobin (569.30 vs 759.01) but faster normalization (8.5 vs 15.92 hours), while in older patients, myoglobin trends favored lightning burns (6 vs 3.25 hours). Pediatric patients with TBSA < 5% had shorter stays (3.83 vs 15.00 days), ICU stays (0.83 vs 13 days), lower initial CPK (508.25 vs 17393.00), lower myoglobin (91.50 vs 10000.00), and quicker CPK normalization (10.00 vs 14.00) in electric burns compared to lightning burns.

**Conclusions:**

Electric burns and lightning burns present similarly in adults. Although initial CPK levels are elevated in lightning burn patients, time taken for CPK to trend down was less than electric burn patients. More research is needed on lightning burn management in a pediatric population.

**Applicability of Research to Practice:**

Lightning burns can be managed similarly to electric burns. Providers ought to expect an increased length of stay in patients who sustain electric burns as well as an extended recovery time for muscular injury.